# mTOR inhibitors are the first-choice therapy for renal angiomyolipomas secondary to tuberous sclerosis

**DOI:** 10.1590/2175-8239-JBN-2023-0077en

**Published:** 2023-07-21

**Authors:** Aline Grosskopf Monich, Mariana Faucz Munhoz da Cunha, Fellype Carvalho Barreto

**Affiliations:** 1 Universidade Federal do Paraná, Departamento de Clínica Médica, Programa de Pós-Graduação em Medicina Interna e Ciências da Saúde, Curitiba, PR, Brazil.; 2 Hospital Universitário Evangélico Mackenzie, Serviço de Nefrologia, Curitiba, PR, Brazil.; 3 Universidade Federal do Paraná, Departamento de Pediatria, Serviço de Nefrologia Pediátrica, Curitiba, PR, Brazil.; 4 Hospital Pequeno Príncipe, Serviço de Nefrologia Pediátrica, Curitiba, PR, Brazil.; 5 Universidade Federal do Paraná, Departamento de Clínica Médica, Serviço de Nefrologia, Curitiba, PR, Brazil.

## Dear Editor

We read with great interest the case report “Endovascular treatment of intrarenal aneurysm bleeding and angiomyolipomas in a patient with tuberous sclerosis and polycystic kidney disease” by Leite et al.^1^, in which embolization was successfully employed. In cases of bleeding, this preemptive interventional and curative treatment is preferable to partial or total nephrectomy, since it preserves the renal parenchyma and kidney function^2^. Currently, however, the preemptive treatment of choice for tuberous sclerosis-related angiomyolipomas is mTOR inhibitors, not embolization^3,4^.

Tuberous sclerosis is an autosomal dominant disease caused by pathogenic variants of the *TSC1* and *TSC2* genes, which encode hamartin and tuberin proteins, respectively. Loss of function of these proteins causes aberrant activation of the mTOR pathway and, consequently, cell cycle modification, changes in the synthesis of proteins, growth factors and lipids, ultimately leading to exacerbated cell proliferation and tumor development and growth^3^. mTOR inhibitors are deemed the first-choice therapy for several manifestations of tuberous sclerosis, including growing renal angiomyolipomas with more than 3 cm in diameter^3^. From a renal standpoint, their potential benefits are: reduction in angiomyolipoma size, lower risk of bleeding due to reduction of intratumoral aneurysms, less need for surgical interventions and preservation of kidney function^3,4^. Cohort studies have also reported a shift in the preventive treatment of renal angiomyolipomas from embolization to mTOR inhibitors^4^.

In agreement with previous studies,^3,5^ preliminary data from an observational cohort study including patients with tuberous sclerosis followed at the Nephrology Service of the Clinics Hospital of the Federal University of Paraná (author’s data) have shown a higher prevalence of angiomyolipomas, renal complications, need of surgical interventions, and of chronic kidney disease in adults compared to pediatric patients. Importantly, our data indicate that a change in the health care provided to these patients is urgently required. The most frequent surgical interventions were still partial and total nephrectomies. Furthermore, despite the clear indications favoring the prescription of an mTOR inhibitor, only a small portion of patients were in use of this medication prior to the first consultation with a nephrologist (Table 1).

**Table 1.** Clinical characteristics of kidney involvement in patients with tuberous sclerosis

	Pediatric patients (<18 years)	Adult patients (≥18 years)
**Patients, *n (%)***	23 (48.9%)	24 (51.1%)
**Age (years)**	9 ± 4	25 ± 7
**Males, *n (%)***	13 (56.5%)	13 (54.1%)
**Renal angiomyolipomas, *n (%)***	6 (26%)	18 (75%)
Bilateral involvement (%)	5 (83.3%)	17 (94.4%)
**Kidney cysts, *n (%)***	7 (30.4%)	7 (29.1%)
**Stage of CKD**		
CKD G2, *n (%)*	0	1 (4.1%)
CKD G3a, *n (%)*	0	3 (12.5%)
CKD G3b, *n (%)*	0	0
CKD G4, *n (%)*	0	0
CKD G5, *n (%)*	0	0
RRT, *n (%)*	0	1 (4.1%)
**SH, *n (%)***	2 (8.6%)	4 (16.6%)
**Use of mTOR inhibitors, *n (%)***	7 (30.4%)	6 (25%)
Neurological indication, *n (%)*	4 (57.1%)	2 (33.3%)
Cardiac indication, *n (%)*	1 (14.2%)	1 (16.6%)
Renal indication, *n (%)*	0	3 (50%)
Dermatologic involvement, *n (%)*	2 (28.5%)	0
**History of kidney complications, *n (%)***	0	7
Retroperitoneal hemorrhage, *n (%)*	0	2 (28.5%)
Partial nephrectomy, *n (%)*	0	1 (14.3%)
Total nephrectomy, *n (%)*	0	2 (28.5%)
Arterial embolization, *n (%)*	0	1 (14.3%)
Death by hemorrhagic shock, *n (%)*	0	1 (14.3%)

CKD: chronic kidney disease; SH: systemic hypertension; RRT: renal replacement therapy.

The case report by Leite et al. highlights that less invasive, renal-sparing surgery provides effective therapy for patients with bleeding from renal AML, one of the most feared complications of tuberous sclerosis, which can result in hemorrhagic shock and death^1,3^. Additionally, we would like to emphasize that tuberous sclerosis patients need regular medical follow-up over lifetime. They must be followed up regularly sincd childhood by a nephrologist, in order to enable early diagnosis of renal involvement, monitoring of its progression and of kidney function, which may allow starting timely mTOR inhibitor treatment, in order to decrease the risk of renal complications and the need for surgery. A multidisciplinary approach, together with strengthening the knowledge about tuberous sclerosis among health care professionals, is crucial for improving the quality of life and survival of these patients.
